# Age Differences in Preferred Methods of Obtaining and Understanding Health Related Information During the COVID-19 Pandemic in Australia

**DOI:** 10.3389/fpubh.2022.912188

**Published:** 2022-07-13

**Authors:** Nathan Williams, Terry Haines, Cylie Williams, Kelly-Ann Bowles, Keith D. Hill

**Affiliations:** ^1^School of Primary and Allied Health Care & National Centre for Healthy Ageing, Monash University, Frankston, VIC, Australia; ^2^Department of Paramedicine, Monash University, Frankston, VIC, Australia; ^3^Rehabilitation Ageing and Independent Living (RAIL) Research Centre, Monash University, Frankston, VIC, Australia

**Keywords:** COVID-19, comprehension, consumer health information, age factors, public health, communication

## Abstract

Methods of communications and the nature of messaging are critically important in influencing public behavior. The COVID-19 pandemic has resulted in major disruptions to all aspects of life globally and has triggered multiple approaches of health messaging to the general public to communicate COVID-19 preventative measures. This study aimed to identify: (1) differences between age groups in the main avenues used by people to obtain COVID-19 related information; and (2) whether age and information sources were associated with correct interpretation of government messaging relating to how people understand or interpret the terms “self-isolation” and “social distancing.” An online survey was conducted in 2020. Participants were aged over 18 years and grouped into age group decades. Differences in sources of COVID-19 information were compared visually between age groups. Logistic regression was used to determine whether age and each of the various methods of communication of COVID-19 information were independently associated with correct response to the self-isolation, or the social distancing statements. There were 3,300 survey respondents 85% female; age sub-groups: 18–29 (7.4%); 30–39 (10.6%); 40–49 (17.6%); 50–59 (22.9%); 60–69 (25.9%); 70–79 (13.9%); and 80+ (1.7%). People accessed public health messaging information from a wide variety of sources that changed as they aged (e.g., older people were more likely to be exposed to COVID-19 information *via* television news programs and less likely via social media platforms). Age was frequently associated with whether the message key terms were interpreted correctly or incorrectly, but in some cases, it promoted more correct responses whereas in others, fewer correct responses. There was no difference between being exposed to COVID-19 information *via* mainstream media, compared with social media, or compared with Government sources of information, in terms of whether COVID-19 messages were interpreted correctly. In order to improve future public health messaging, there is a need for multiple avenues of communication to meet the needs and preferences across and within age groups. Further investigation is warranted into the clarity of the content and method of delivery of public health messages, to ensure optimal understanding of public health messages by vulnerable populations and across the community.

## Introduction

The COVID-19 pandemic has resulted in major disruptions to all aspects of life globally, since first detected in December 2019 (1–6). The disruptions have resulted from both the direct impact of the virus on the health of populations, especially vulnerable populations such as older people, people with disabilities, and first Nations people (7–9); and the added impact of government responses and restrictions imposed aiming to control spread and impact of the virus.

The nature of state and national responses in Australia and around the world have varied markedly, and changed substantially over time (10, 11). With changing health messages, it is imperative that key elements of messaging, such as changes in restrictions, changes to personal actions (e.g., differing advice regarding wearing of face masks in different environments) and the rationale for these public health responses are conveyed widely, quickly and clearly. There are many avenues for communicating these public health messages to the wider community, with some more likely to have better reach and viewing in differing segments of the community (12). Some of the common avenues for communicating these messages relating to the COVID-19 pandemic include regular (often daily) television media conferences by state and national politicians, television news programs, newspapers, social media (including Facebook, Twitter), and video (13, 14). How these public health messages are framed can also influence their reach and impact, with one recent study highlighting that the most effective methods of framing COVID-19 messages through the World Health Organization had doubled the engagement than the least well performing methods of framing messages (15).

Throughout the COVID-19 pandemic, evidence has highlighted that older people are at increased risk of experiencing severe symptoms of disease and of negative health outcomes, including increased hospitalization and a higher mortality rate (16–18). Therefore, knowing where people of different ages access their COVID-19 information and analyzing their understanding of that information is relevant and important, and it could save lives.

There is existing evidence that there are some differences between age groups across the lifespan about how public health information is accessed and used in general (19). There are also differences in engagement when comparing different social media modes of disseminating public health information (20). However, there is relatively little research relating to differences between age groups and health messaging specifically relating to the COVID-19 pandemic. A large international survey (*n* = 17,287, 76% from the United States of America) identified differences in the impacts of COVID-19 between age groups (18–31; 32–44; 45–64; 65+), as well as differences in compliance with public health messaging regarding measures such as social distancing and self-isolation (lower compliance in younger age groups) (21). The paper reports different recommendations by age group to improve messaging by age group, but does not consider the avenues of public health messaging. A large web-based survey (*n* = 125, 508) identified that respondents over 65 years of age were more likely to have COVID-19–related concerns, engage in precautionary behaviors, such as wearing a face mask, and were less willing to return to previous activities they engaged in (22). Differences in health literacy between younger and older people have also been reported (23) that may influence interpretation, perception of relevance, and adherence to public health messaging. Therefore, there is a need to further investigate how people of different ages are sourcing and understanding such health-related messaging.

Burke et al. (24) found that during Hurricane Katrina in the United States of America in 2005, differences in age were found to be of importance for the use of television and radio warning messages. Therefore, there is a need to investigate whether this is still the case in a more recent significant public health event—COVID-19. Is there still a relationship between increasing age and information source being used? Or is there now more of a reliance on social media or other avenues of communication?

Twitter, for example, has been noted to be a source of misinformation in the past, such as during the 2014−2016 Ebola outbreak in West Africa, where Twitter was used to spread misinformation and false cures (25). During the COVID-19 pandemic, Twitter has also largely become an outlet for the spread of misinformation, with one study showing that Twitter posts with false claims propagate faster than other claims (26). Therefore, it is appropriate to investigate the extent to which people in Australia are using Twitter as a COVID-19 information source, and further, which other social media and information sources are being used? Are the information sources being used leading to further misinformation and confusion, or are the public health messages being interpreted as intended?

It was hypothesized that older people would prefer more mainstream media sources (newspapers, television and radio) and that younger people would prefer more social media sources (Twitter, Instagram, TikTok) and word of mouth. It was also hypothesized that there would be significant differences in people's understanding of social distancing and self-isolation according to their age and information source used, but to what degree (positively or negatively) was uncertain.

The findings of this research would inform future public health messaging, both during the COVID-19 pandemic and for future public health issues. It would inform relevant parties as to where people of different ages get their public health information from, and it would inform them of whether or not age is an important consideration when developing such public health messaging. There are also potential cost benefits, given that the knowledge of which information sources are being used by whom could lead to the production of less unnecessary messaging materials, and therefore reducing costs. The findings could also be used to inform future, more targeted messaging, particularly to certain age groups or users of a particular form of media.

To date, the issue of differences between age groups in preferred approaches to receiving COVID-19 public health messaging has received little research attention. This study aimed to identify: (1) differences between age groups in the main avenues used to obtain COVID-19 related information; and (2) whether age and information sources were associated with correct interpretation of government messaging relating to self-isolation and social distancing.

## Materials and Methods

This study utilized a cross-sectional design to survey issues related to knowledge, attitudes and preferred communication strategies of adult Australians related to the COVID-19 pandemic. The project was approved by Monash University Human Research Ethics Committee (project ID: 24040). The CHERRIES (CHEcklist for Reporting Results of Internet E-Surveys) framework was used to guide the reporting of survey results (27).

### Participants

Participants had to be citizens or permanent residents of Australia and they had to be aged 18 years and over, in order to be eligible to participate in the survey. Participants were excluded if their provided postcode was not clearly from within Australia and if they did not answer the questions regarding age, social distancing and self-isolation i.e., the most relevant questions to this analysis. These respondents were included in the study, but if they did not answer the relevant questions (age, information source, social distancing, self-isolation) then they were excluded from our analysis.

A priori power analysis was used to guide the sampling framework for this research. *Post-hoc* power analysis identified that if we wanted to compare responses between participants over the age of 60 (*n* = 1,312) with those less than this age (*n* = 1,988) on a dichotomous outcome, we have 80% power to detect an increase in proportion in the older adult group 0.05 assuming a baseline proportion in the younger adult group of 0.40 (i.e., 0.40 younger vs. 0.45 older).

The convenience sample of survey respondents were recruited through a range of promotional activities and modes, primarily social media, including Facebook advertising, Twitter, and Instagram (targeting Australians and age >18 years). The promotional avenues included a link to the survey, developed using Qualtrics® software (Qualtrics, Provo, UT, United States). Recruitment occurred in the early stages of the COVID-19 pandemic in Australia (April 1, 2020—June 3, 2020).

### Survey and Associated Procedures

The survey was developed by a large interdisciplinary team with expertise in health, primary care, ageing, and qualitative and quantitative research, that included this study authors. The overall survey involved an extensive data-set of 97 questions (see Appendix 1 for questions relevant to this analysis) with a subset of the survey being utilized to address the aims of this study. Survey question items were developed in the following domains:

i) Demographic questions, including age, gender, state, and employment categories (Table 1). Most relevant to this analysis, participants were asked to select their age bracket: <30 years, 30–39 years, 40–49 years, 50–59 years, 60–69 years, 70–79 years, and 80 years or more.
ii) People's perceptions of the extent to which they had been exposed to information about COVID-19 in the previous month from a list of different information sources. Namely: Newspapers, Radio, Television - news programs, Television - other programs, Facebook feeds, Facebook private groups, Instagram, Twitter, TikTok, Online blogs, Podcasts, General browsing on the internet, Australian Government “Coronavirus Australia” App, Other webpages and resources specifically prepared by the Australian Government, Workplace, Friends and family, and Other sources (please specify). We asked participants whether they had been exposed to COVID-19 information from each source on the following scale: Not at all, to a small extent, to a moderate extent, or to a great extent.
iii) Participant knowledge of the concepts of social distancing and self-isolation related to COVID-19. We asked participants whether they agreed, disagreed or were unsure (Yes/ No/ Unsure responses) that examples we provided were included in these concepts. We drew examples from the definitions of these concepts adopted by the Australian Government and published on the Australian Government (28) and Prime Minister (29) websites at the time of the survey (Appendix 2). We also included examples of distractors (untrue) statements relative to the Australian Government definitions. Using these same definitions, Yes/ No/ Unsure responses were then converted into either correct or incorrect responses for data analysis (see Tables 2, 3).

**Table 1:** Sample demographics across age groups.

**Age**	**Full Sample, *n* (%) = 3,300 (100)**	**Under 30, *n* (%) = 243 (7.4)**	**30–39, *n* (%) = 351 (10.6)**	**40–49, *n* (%) = 581 (17.6)**	**50 −59, *n* (%) = 757 (22.9)**	**60 −69, *n* (%) = 855 (25.9)**	**70–79, *n* (%) = 457 (13.9)**	**≥80, *n* (%) = 56 (1.7)**
**Gender—*****n*** **(%)**								
Male Female Non-binary/ Other	476 (14.4) 2,815 (85.3) 9 (0.3)	37 (15.2) 206 (84.8) 0 (0.0)	23 (6.6) 325 (92.6) 3 (0.9)	53 (9.1) 526 (90.5) 2 (0.3)	103 (13.6) 652 (86.1) 2 (0.3)	140 (16.4) 713 (83.4) 2 (0.2)	99 (21.7) 358 (78.3) 0 (0.0)	21 (37.5) 35 (62.5) 0 (0.0)
**Marital status—*****n*** **(%)**								
Married/de facto Widowed Divorced Separated, not divorced Single or never married Partner/not living together Did not answer	2,161 (65.5) 188 (5.7) 375 (11.4) 119 (3.6) 394 (11.9) 22 (0.7) 41 (1.2)	96 (39.5) 0 (0.0) 0 (0.0) 0 (0.0) 138 (56.8) 6 (2.5) 3 (1.2)	289 (82.3) 0 (0.0) 4 (1.1) 11 (3.1) 41 (11.7) 1 (0.3) 5 (1.4)	424 (73.0) 9 (1.6) 36 (6.2) 23 (4.0) 78 (13.4) 4 (0.7) 7 (1.2)	523 (69.1) 20 (2.6) 101 (13.3) 35 (4.6) 61 (8.1) 3 (0.4) 14 (1.9)	537 (62.8) 53 (6.2) 158 (18.5) 35 (4.1) 59 (6.9) 4 (0.5) 9 (1.1)	267 (58.4) 80 (17.5) 73 (16.0) 15 (3.3) 16 (3.5) 3 (0.7) 3 (0.7)	25 (44.6) 26 (46.4) 3 (5.4) 0 (0.0) 1 (1.8) 1 (1.8) 0 (0.0)
**Low Income Household—*****n*** **(%)**								
Yes	470 (14.2)	38 (15.6)	39 (11.1)	70 (12.1)	97 (12.8)	138 (16.1)	81 (17.7)	7 (12.5)
**Govt. Pension/ Payment—*****n*** **(%)**								
Yes	848 (25.7)	49 (20.2)	40 (11.4)	75 (13.0)	72 (9.5)	240 (28.1)	327 (71.6)	45 (80.4)
**Have a chronic health condition—*****n*** **(%)**								
Yes	1,450 (43.9)	81 (33.3)	102 (29.1)	213 (36.7)	315 (41.6)	436 (51.0)	263 (57.6)	40 (71.4)
**Employment status—*****n*** **(%)**								
Full time Part time Casual Self-employed Unpaid Looking for work Retired Student Seeking employment Other Unable to work due to health	1,008 (30.6) 557 (16.9) 203 (6.2) 239 (7.2) 152 (4.6) 108 (3.3) 806 (24.4) 140 (4.2) 54 (1.6) 7 (0.2) 26 (0.8)	69 (28.4) 25 (10.3) 36 (14.8) 3 (1.2) 7 (2.9) 15 (6.2) 0 (0.0) 83 (34.2) 3 (1.2) 0 (0.0) 2 (0.8)	136 (38.8) 98 (27.9) 18 (5.1) 21 (6.0) 41 (11.7) 9 (2.7) 0 (0.0) 23 (6.6) 3 (0.9) 2 (0.6) 0 (0.0)	276 (47.5) 132 (22.7) 41 (7.1) 49 (8.4) 26 (4.5) 21 (3.6) 1 (0.2) 16 (2.8) 12 (2.1) 0 (0.0) 7 (1.2)	357 (47.2) 158 (20.9) 50 (6.6) 65 (8.6) 30 (4.0) 35 (4.6) 30 (4.0) 12 (1.6) 12 (1.6) 1 (0.1) 7 (0.9)	159 (18.6) 123 (14.4) 46 (5.4) 75 (8.8) 31 (3.6) 26 (3.0) 360 (42.1) 5 (0.6) 17 (2.0) 4 (0.5) 9 (1.1)	10 (2.2) 21 (4.6) 12 (2.6) 23 (5.0) 15 (3.3) 2 (0.4) 366 (80.1) 1 (0.2) 6 (1.3) 0 (0.0) 1 (0.2)	1 (1.8) 0 (0.0) 0 (0.0) 3 (5.4) 2 (3.8) 0 (0.0) 49 (87.5) 0 (0.0) 1 (1.8) 0 (0.0) 0 (0.0)

**Table 2:** Factors (age and information sources) associated with correct responses to the questions regarding self-isolation.

**Self-isolation means that you**	**Correct response (% correct)**	**Age** **Odds Ratio (95% CI)**	**News-paper Odds Ratio (95% CI)**	**TV Other Odds Ratio (95% CI)**	**FBF Odds Ratio (95% CI)**	**FBP Odds Ratio (95% CI)**	**Twitter Odds Ratio (95% CI)**	**Blogs Odds Ratio (95% CI)**	**Internet browsing Odds Ratio (95% CI)**	**Govt. app Odds Ratio (95% CI)**	**Govt. websites Odds Ratio (95% CI)**	**Workplace Odds Ratio (95% CI)**	**Friends & family Odds Ratio (95% CI)**
Be the only person who lives at your place of residence	No (79.19%)	0.76 (0.71–0.82)	1.09 (1.00– 1.19)	0.85 (0.77– 0.94)		0.90 (0.81–1.00)	1.22 (1.01–1.47)	1.25 (1.03–1.52)					
Are not to let visitors into your house	Yes (96.45%)	0.73 (0.62–0.86)											
Are not to go out into the community for any reason	No (16.57%)	1.13 (1.05– 1.22)						1.31 (1.11– 1.56)		0.87 (0.78 – 0.96)			
Only go out into the community if wearing a face mask	Yes (9.75%)	1.12 (1.01 – 1.23)	0.89 (0.79 – 1.00)		0.86 (0.74 – 0.99)				1.45 (1.24–1.70)		0.84 (0.72–0.96)	1.13 (1.00– 1.27)	
Can go out into the community if purchasing food or other necessities, but only if you are wearing a face mask	No (84.52%)	0.89 (0.83–0.96)							0.87 (0.77 −0.98)		1.14 (1.01–1.28)		
Can go out into the community if seeking medical attention, but only if you are wearing a face mask	Yes (38.32%)	0.86 (0.82–0.91)							1.14 (1.04 −1.25)	1.11 (1.03–1.20)			
Can go out into the community if seeking medical attention. you can wear a face mask if you have one but do not have to if you do not have one	No (58.70%)	0.94 (0.88–0.99)						0.78 (0.68–0.89)				1.15 (1.07–1.23)	

*Correct Answer = 1, Incorrect Answer = 0 CI, Confidence Interval; FBF, Facebook Feeds; FBP, Facebook Private; Groups Results only included in table when p < 0.05 for each of Age or Information Source. Odds Ratio >1 indicates increased likelihood of correct response TV News, Radio, Instagram, TikTok and Podcasts not included in this table as they were not statistically significant for these questions*.

**Table 3:** Factors (age and information sources) associated with correct responses to the questions regarding social distancing.

**Social distancing means that you…**	**Correct response (% correct)**	**Age odds ratio (95% CI)**	**Radio odds ratio (95% CI)**	**TV other odds ratio (95% CI)**	**Facebook feeds odds ratio (95% CI)**	**Podcasts odds ratio (95% CI)**	**Govt. app odds ratio (95% CI)**	**Govt. websites odds ratio (95% CI)**	**Friends & family odds ratio (95% CI)**
Should stay at home as much as possible	No (10.40%)	1.34 (1.22 −1.48)							
Should stay at home if feeling unwell	Yes (96.40%)	0.77 (0.66–0.91)							
should restrict your physical contact to just your family & friends	No (40.77%)	0.94 (0.89 −1.00)							0.87 (0.79–0.95)
Should stay 1.5 m away from other people at all times	No (7.70%)						0.84 (0.72–0.98)		
Should stay 1.5 m away from other people where possible	Yes (79.99%)		1.17 (1.06–1.29)			0.84 (0.74–0.96)		0.86 (0.78–0.96)	
Can't exercise outdoors	No (94.71%)	0.76 (0.66–0.87)		0.83 (0.69–0.99)			0.84 (0.72 −0.99)		
Can't exercise outdoors in groups of over 10 people, while staying 1.5 m away from these people while doing this	No (27.59%)	1.09 (1.02–1.16)		0.89 (0.82 – 0.98)					
Can only exercise outdoors with people who live at your residence	No (39.68%)	1.11 (1.05–1.18)			0.90 (0.83–0.98)				

*Correct Answer = 1, Incorrect Answer = 0 Results only included in table when p < 0.05 for each of Age or Information Source. Odds Ratio >1 indicates increased likelihood of correct response Newspapers, TV News, Facebook Private Groups, Instagram, Twitter, TikTok, Blogs, Internet Browsing and Workplace not included in this table as they were not statistically significant for these questions*.

The overall survey was estimated to take approximately 18 min (median time for completion). The survey utilized forced or requested responses to reduce missing data, although the respondents could exit the survey at any time (completed responses were saved to the time of exit). A brief explanatory statement at the start of the survey described the purpose of the survey, ethics committee approval, participant anonymity, and indicated that proceeding with the survey indicated implied consent to participate. No incentives were offered for completing the survey. Respondents completing the survey were not identifiable.

### Data Collection

This was a completely online survey that was advertised through social media platforms and through local health and community networks. Participants accessed the survey online and the survey was completely self-administered. The data collection was fully automated using the Qualtrics® program. Participants completed the survey using their own phones or computers in their own locations. There was no central in-person data collection process undertaken.

### Data Analysis

Survey data from the Qualtrics® program was downloaded as an excel file, and imported to Stata 15 (StataCorp, College Station, TX, United States), to analyse data. Descriptive statistics means and standard deviation, median [IQR] or frequencies (%) were used for describing the sample characteristics, depending on the nature of the question and data distribution. Frequency and percentage responses were used to report data relating to the survey questions of interest, with data broken down into the following age groups: <30 years; 30–39; 40–49; 50–59; 60–69; 70–79; and ≥80 years.

Responses to all questions relating to the aims were graphed comparing responses across the age groups. To address aim 2, logistic regression was used to determine whether age, and each of the various methods of communication of COVID-19 information (e.g., newspaper, television, radio, Facebook, Twitter, etc.) were independently associated with correct response to the self-isolation, or the social distancing statements. Statistical significance was set at *p* < 0.05. Results were expressed as odds ratios with 95% confidence intervals, where 0 = Incorrect response and 1 = Correct response (see Tables 2, 3).

## Results

A total of 3,415 people responded to the survey; 95 were removed due to postcode errors where it could not be certain that the respondents were living in Australia. A further 20 respondents were removed as there was insufficient data provided in order to answer the research questions (did not respond to age, social distancing, self-isolation questions). Therefore, 3,300 respondents formed the full sample.

The 50–59 (*n* = 757, 22.9%) and the 60–69 age groups (*n* = 855, 25.9%) were the groups with the highest proportion of respondents, while the lowest proportion of responses were from the youngest (<30 years, *n* = 243, 7.4%), and oldest (≥80, *n* = 56, 1.7%) sub-groups of the sample. Table 1 reports the main sample demographics. Overall, 85% of respondents were female, 65% were married or de facto, and 43% reported having one or more chronic health condition. The majority were in full time, part time or casual employment (53.7%) or were self-employed (7.2%) or retired (24.4%). There were a number of differences in the demographic profile variable between the different age groups.

**Figure 1:**
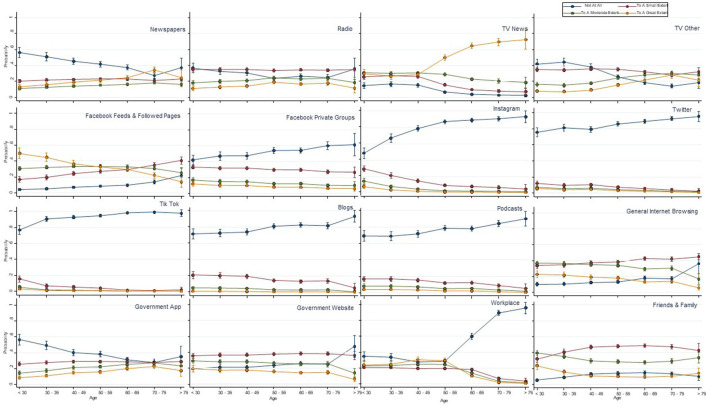
Sources of exposure to COVID-19 information across age groups (probability of each response with 95%CI).

There were some sources of information access that differed substantially between age groups and some that appeared similar between all groups (Figure 1). The main source of information with increasing levels of access by older age groups was television news from age 40 and above. Sources of information with lowest levels of access by older age groups included multiple social media avenues (Facebook, Instagram, Twitter, TikTok, blogs), podcasts and the workplace.

**Figure 2:**
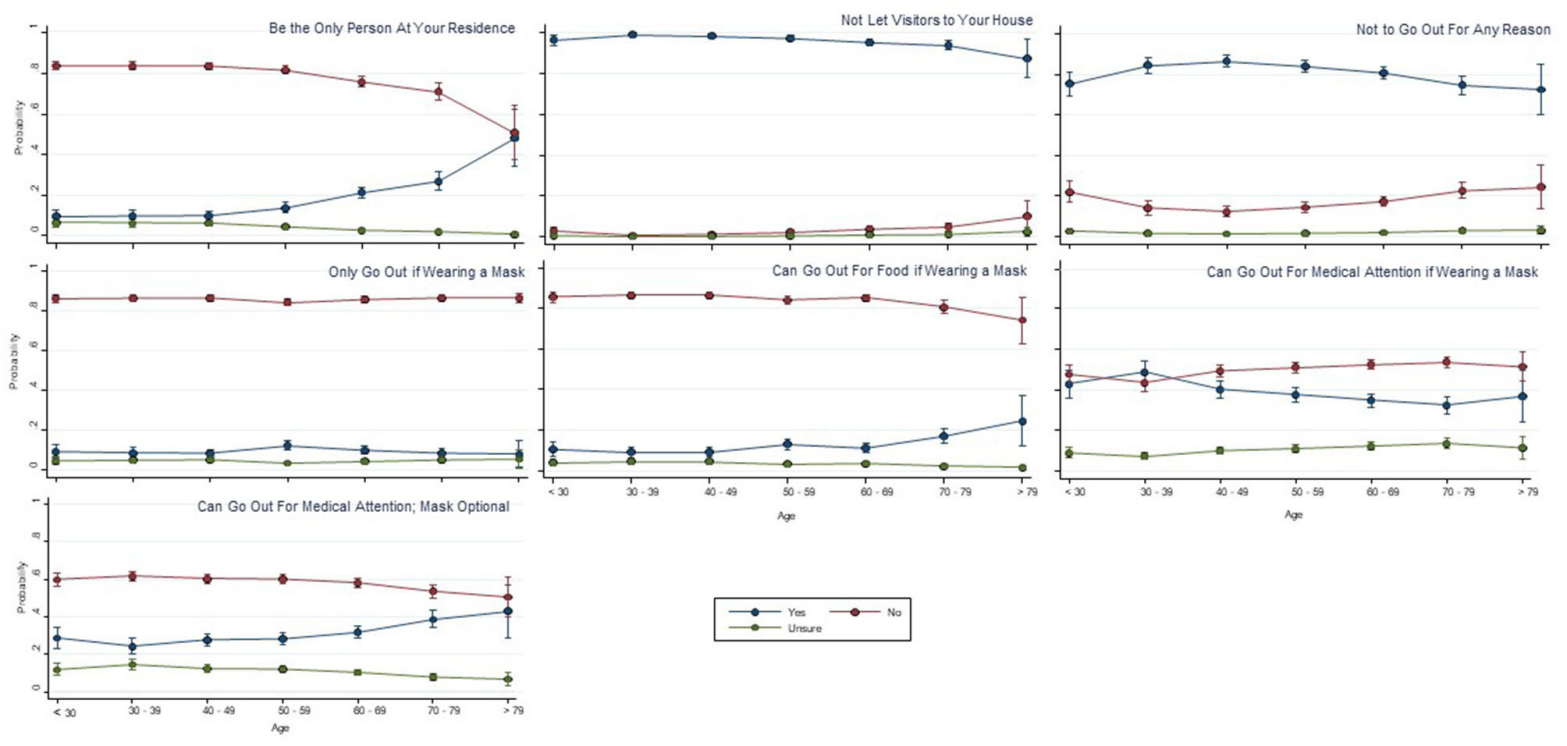
Self-isolation means that you … (probability of each response with 95%CI).

**Figure 3:**
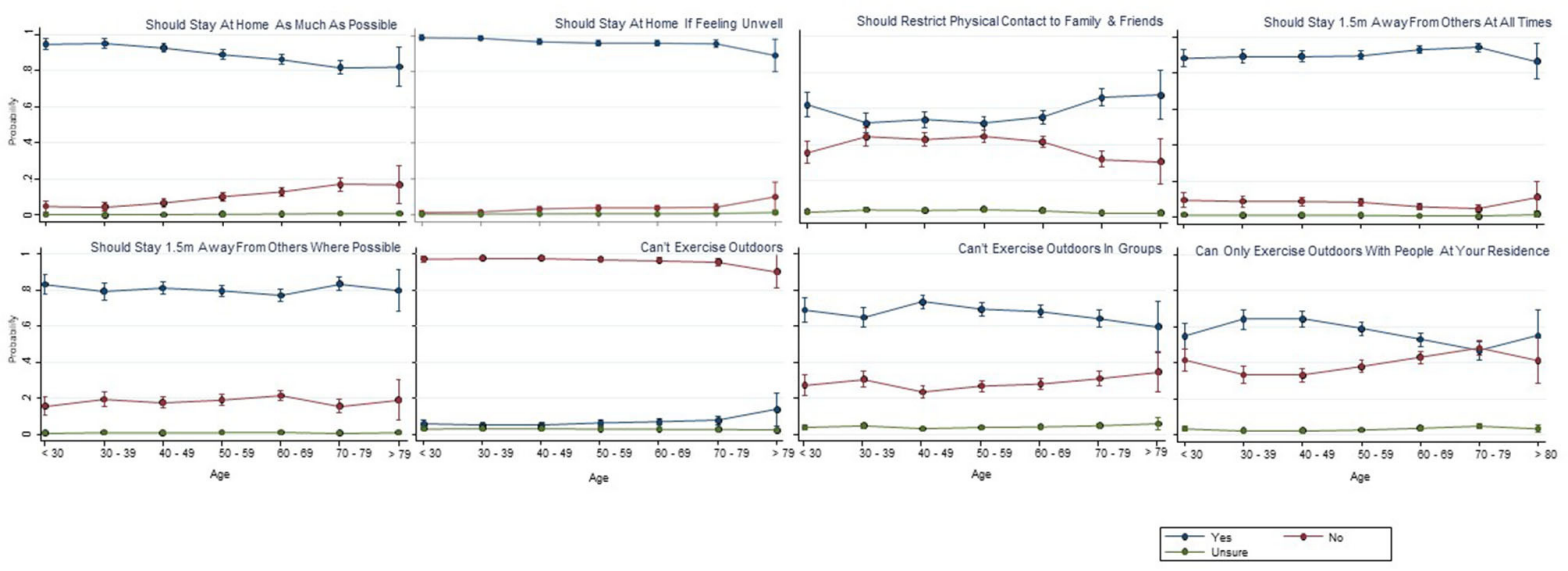
Social distancing means that you … (probability of each response with 95%CI).

Figures 2, 3 show the responses by age group and type of information access to various statements relating to the meaning of key terms of self-isolation and social distancing respectively. Visual analysis of these graphs for the self-isolation statements (reporting yes/no responses, irrespective of correct response) highlights increasing proportions of the three older age groups answering yes to statements that self-isolation means: (1) being the only person at your residence; (2) can go out for medical attention, mask optional; and (3) can go out for food if wearing a mask; and there was a small reduction in the proportion in older age groups answering yes to the social distancing statements of “Should stay at home as much as possible” and “Can't exercise outdoors in groups”; and an increase in the proportion of the older age groups answering yes to “Should restrict physical contact to family and friends.” In several of the figures, particularly for the social distancing statement, the oldest age group (>79 years) appeared to respond differently to the trend of other age groups (e.g., can only exercise outdoors with people at your residence), although these differences just in this oldest age group should be interpreted with caution given the small sample size in that group (1.7% of overall sample).

Tables 2, 3 show the results of the logistic regression for each question relating to both self-isolation and social distancing, with respect to age and each of the information sources. Age and information sources were included in the Tables if *p* < 0.05, represented as an odds ratio and with 95% confidence intervals. For these analyses, data were converted from yes/ no/ unsure responses into either correct or incorrect responses (as shown in Tables 2, 3). A score of 1 indicated a correct response and a score of 0 indicated an incorrect response. An odds ratio >1 indicates an increased likelihood of answering the question correctly with increasing age and conversely, an odds ratio <1 increased likelihood of answering the question incorrectly with increased age. The percentage of correct responses varied substantially for the various self-isolation (9.8–96.5% correct) and social distancing questions (7.7–96.4% correct). There was a significant association between age and correct responses in the majority of responses to the self-isolation and social distancing questions, but no clear overall pattern of being more or less likely to respond correctly (Tables 2, 3). Logistic regression results indicated significant differences for various information sources to the different social distancing and self-isolation questions, but again, there was no clear pattern relating to any specific information source and greater accuracy of responses.

There was no clear trend in correct or incorrect interpretation based on whether people used traditional mainstream media (Television, radio, newspapers) compared with both social media (Facebook, Twitter, Instagram, TikTok) and Government sources (websites, app).

## Discussion

Public health practitioners and policy makers who want to communicate messages to middle and older age groups need to utilize television news programs, as this is where these people are getting the greatest amount of exposure to COVID-19 information. Older people have very little exposure to social media sources (e.g., Instagram, Tik Tok), therefore strategies to target older people should not focus on these social media options. For some social media, virtually no one was using them, despite the investment in using these media by public health officials. For example, Twitter was seldomly used by our participants, and yet, State Governments released daily updates on Twitter (30). However, there may be other considerations for this, such as low cost of constructing a daily Twitter post, that may also influence the decision by policy makers to use Twitter often. It is also important to have clear messages about what the public health instructions are, because the results from our investigation indicate that there was a large amount of confusion, and there did not seem to be a clear pattern as to whether older people were interpreting the messages any more or less correctly than younger people. Although there was confusion across the board, the areas that are arguably the most concerning are the concepts relating to people not leaving their homes for any reason, including for possible health concerns requiring medical attention. A potential issue with a large and often changing public health response such as COVID-19, is that there might be such a volume of new information and new instructions that are released, it may be unreasonable to expect that the public will be able to understand everything, and that the most parsimonious messaging approach is more likely to be needed.

Our research is concordant with previous literature in related fields that has investigated the use of television by older people as an information source for public health messaging. However, our study also indicates a lack of use of social media avenues by older people, and that people were generally confused by the messaging, regardless of their age or information source used. Similarly to our investigation, Burke et al. (24) found an increased use in television as age increased, during Hurricane Katrina in 2005. In contrast, they also found increased use of radio by older people, which was not evidently the case in our study. Similarly to our study, Moreno, Fuentes and Navarro (31) found that three of the four most commonly used information sources in Spain in 2020, were mainstream news media. Our study expands upon this by also investigating those people's ages. In contrast, WhatsApp was the second most commonly used information source (31), which was not investigated in our study. However, similar WhatsApp messages might have been captured in this research when considering responses from friends and family. Concordantly with our study, Daoust (32) found that older and younger people's attitudes and behaviors were similar during the COVID-19 pandemic, with no clear pattern in relation to increased age.

Limitations of this research include the rapidly changing nature of living during a pandemic and the impact that may have had on changes in perceptions and behavior, and the ultimate relevance of the research findings. For example, it is possible that there has been a change in which information sources are most commonly used by different age groups since the survey was conducted in 2020. In addition, the interpretations of social distancing and self-isolation have likely changed and changed again, as different rules and lockdown restrictions have been implemented and altered throughout the course of the COVID-19 pandemic. People may now have a greater understanding of what those terms mean, given that more time has now passed since the beginning of the pandemic. However, despite these factors, this research informs the importance of clarity of messaging at the very beginning of a pandemic or public health issue, regardless of whether there is a need to change the message thereafter. Furthermore, this research suggests the benefit of communicating any changes in the message (e.g., new lockdown restrictions) via a variety of sources, with consideration to people of different ages. In addition, given that the recruitment and the completion of the survey were done using technology (i.e., online and through social media), there may have been issues with access and inclusion of some participants. Those who were unable to use and access information technology, the internet and social media and those with certain health conditions and disabilities that prevented them from accessing the survey, may have been inadvertently excluded from participating. An additional limitation is that the majority of respondents were female, which would have skewed any attempt at assessing differences in responses based upon gender. Furthermore, some items which had very low percentages of correct responses may have had issues with the wording or clarity of the question, resulting in some more cautious participants potentially selecting the more restricted (and therefore technically incorrect) response.

In this research, we have sought to understand how older people engage with and understand key concepts regarding COVID-19, however there are additional vulnerable population groups other than older people. These vulnerable groups might be defined by factors such as language barriers, socioeconomic disadvantage, a limited ability to access healthcare and health information, and the presence of health conditions. Further research is needed to understand how these other vulnerable populations access their COVID-19 information and general public health messaging.

## Conclusion

Overall, this research demonstrates that people prefer to get their public health messaging from a wide range of information sources, which change as they age, most obviously regarding the use of television news and various forms of social media. Age is a significant factor in whether that message is interpreted correctly or incorrectly, but that interpretation is not consistently more or less likely to be correct or incorrect with increasing age. Generally, both older and younger people were confused with their responses despite their information source used, evidenced by inconsistent correct and incorrect responses across the board, with no clear trend, which potentially speaks to the issue being the clarity of the message itself. There was no clear benefit of using one information source compared with any other. In order to provide benefit to future public health messaging, further research is needed on how to provide further clarity in how that messaging is interpreted. Further investigation is warranted into the clarity of the content and the method of delivery of public health messages, with a consideration of the age of the intended audience, regardless of the information source that is used.

## Data Availability Statement

The raw data supporting the conclusions of this article will be made available by the authors, without undue reservation.

## Ethics Statement

This study involving human participants was reviewed and approved by the Monash University Human Research Ethics Committee. Participants selected that they provided their informed consent at the commencement of the online survey to participate in this study.

## Author Contributions

TH, CW, KH, and K-AB conceptualized and designed the study, designed the survey questions, and organized the recruitment of participants. CW and K-AB collated and prepared the raw data for data analysis. NW performed the data analysis, created the figures and tables and suggestions for revision were incorporated. NW and KH drafted the first version of the manuscript with input and guidance from TH and all other authors throughout. All authors provided critical review of the draft and suggestions for revision. All authors contributed to the article and approved the submitted version.

## Funding

This work was funded by Grant number is RRCSA000014.

## Conflict of Interest

The authors declare that the research was conducted in the absence of any commercial or financial relationships that could be construed as a potential conflict of interest.

## Publisher's Note

All claims expressed in this article are solely those of the authors and do not necessarily represent those of their affiliated organizations, or those of the publisher, the editors and the reviewers. Any product that may be evaluated in this article, or claim that may be made by its manufacturer, is not guaranteed or endorsed by the publisher.
