# Supplementary material for: Age Differences in Preferred Methods of Obtaining and Understanding Health Related Information During the COVID-19 Pandemic in Australia
Source: Front Public Health. 2022 Jul 13;10:912188. doi: 10.3389/fpubh.2022.912188 (PMC9326317; doi:10.3389/fpubh.2022.912188)
Supplement: Supplementary file 1 [file Data_Sheet_1.pdf]

Appendix 1 – Relevant Survey Questions for Completion by Participants

- Please select your age bracket

- ○ Less than 30 years (1)
- ○ 30-39 years (2)
- ○ 40-49 years (3)
- ○ 50-59 years (4)
- ○ 60-69 years (5)
- ○ 70-79 years (6)
- ○ 80 years or more (7)

There are many potential sources of information about COVID-19 and how we should respond to it.

Please indicate the extent to which you have been exposed to information about COVID-19 in the past month from... (Every response must be clicked to progress).

	Not at all (1)	To a small extent (2)	To a moderate extent (3)	To a great extent (4)
Newspapers (1)	○	○	○	○
Radio (2)	○	○	○	○
Television - news programs (3)	○	○	○	○
Television - other programs (4)	○	○	○	○
Facebook feeds (5)	○	○	○	○
Facebook private groups (6)	○	○	○	○
Instagram (7)	○	○	○	○
Twitter (8)	○	○	○	○
TikTok (9)	○	○	○	○
Online blogs (10)	○	○	○	○
Podcasts (17)	○	○	○	○
General browsing on the internet (11)	○	○	○	○
Australian Government "Coronavirus Australia" App (16)	○	○	○	○
Other web-pages and resources specifically prepared by the Australian Government (12)	○	○	○	○
Workplace (13)	○	○	○	○

Friends and family (14)	○	○	○	○
Other sources (please specify) (15)	○	○	○	○

- For the next set of questions, we are interested in understanding what people think it means to "self-isolate" for 14 days.
Please indicate if you think that to "self-isolate" means that you...

	Yes (1)	No (2)	Unsure (3)
... be the only person who lives at your place of residence (1)	○	○	○
... are not to let visitors into your house (2)	○	○	○
... are not to go out into the community for any reason (3)	○	○	○
... only go out into the community if wearing a face mask (4)	○	○	○
... can go out into the community if purchasing food or other necessities, but only if you are wearing a face mask (5)	○	○	○
... can go out into the community if seeking medical attention, but only if you are wearing a face mask (6)	○	○	○
... can go out into the community if seeking medical attention. You can wear a face mask if you have one but do not have to if you do not have one. (7)	○	○	○

- For the next set of questions, we are interested in understanding what people think it means to exercise "social distancing".
Please indicate what you think it means to exercise "social distancing".

	Yes (1)	No (2)	Unsure (3)
That you should stay at home as much as possible (1)	○	○	○
That you should stay at home if feeling unwell (2)	○	○	○
That you should restrict your physical contact to just your family and friends (3)	○	○	○
That you should stay at least 1.5 metres away from other people at all times (4)	○	○	○
That you should stay 1.5 metres away from other people where possible (5)	○	○	○
That you can't exercise outdoors (6)	○	○	○
That you can't exercise outdoors in groups of more than 10 people, while staying 1.5 metres from these people while doing this (7)	○	○	○
That you can only exercise outdoors with people who live at your residence (8)	○	○	○

Appendix 2 – Government Messages on Social Distancing and Self-Isolation that Informed Which Answers Are Correct and Incorrect:

This research used the information from the Australian Government(28) and Australian Prime Minister(29) websites during March – June 2020. The below descriptions taken from these websites, informed the decision of whether the respondents had correctly or incorrectly interpreted the public health messaging at the time (as per Tables 2 and 3):

Social Distancing:

Social distancing is one way to help slow the spread of viruses such as COVID-19.

Social distancing includes staying at home when you are unwell, avoiding large public gatherings if they're not essential, keeping a distance of 1.5 metres between you and other people whenever possible and minimising physical contact such as shaking hands, especially with people at higher risk of developing serious symptoms, such as older people and people with existing health conditions.

There's no need to change your daily routine, but taking these social distancing precautions can help protect the people in our community who are most at risk.

Self-Isolation:

If you have been diagnosed with COVID-19, you must stay at home to prevent it spreading to other people. You might also be asked to stay at home if you may have been exposed to the virus. Staying at home means you:

- Do not go to public places such as work, school, shopping centres, childcare or university
- Ask someone to get food and other necessities for you and leave them at your front door
- Do not let visitors in — only people who usually live with you should be in your home

You do not need to wear a mask in your home. If you need to go out to seek medical attention, wear a surgical mask (if you have one) to protect others. You should stay in touch by phone and on-line with your family and friends.
